# Complete Genome Sequence of an Emerging Genotype of Tobacco Streak Virus in the United States

**DOI:** 10.1128/genomeA.01138-14

**Published:** 2014-11-06

**Authors:** Chellappan Padmanabhan, Shan Gao, Rugang Li, Shouan Zhang, Zhangjun Fei, Kai-Shu Ling

**Affiliations:** a Department of Agriculture, Agricultural Research Service, U.S. Vegetable Laboratory, Charleston, South Carolina, USA; b Boyce Thompson Institute for Plant Research, Cornell University, Ithaca, New York, USA; c Tropical Research & Education Center and Department of Plant Pathology, University of Florida, IFAS, Homestead, Florida, USA; d Department of Agriculture, Agricultural Research Service, Robert W. Holley Center for Agriculture and Health, Ithaca, New York, USA

## Abstract

We report here the complete genome sequence of an emerging genotype of tobacco streak virus (TSV) infecting zucchini squash in Florida (TSV_FL13-07), obtained using deep sequencing of short RNAs (sRNAs) and validation by Sanger sequencing. TSV_FL13-07 shares only <90% sequence identity in all three genomic RNAs to several known U.S. isolates.

## GENOME ANNOUNCEMENT

Ilarviruses belong to the family *Bromoviridae* and have a nonenveloped quasispherical virion with a segmented positive single-stranded RNA (ssRNA) genome, a 3′ tRNA-like structure, and a 5′ cap. Tobacco streak virus (TSV), a type member of *Ilarvirus*, has a wide host range infecting many plant species in >30 families worldwide. TSV is transmitted by thrips and can be seed transmissible. In April 2013, a zucchini type of summer squash, “Senator,” grown in an 80-acre field in Homestead, FL, displayed unusual severe mosaic and epinasty symptoms, with an approximately 5% infection rate, and with one corner of the field near 100% infection. Screening with serological tests to 13 commonly known viruses in cucurbits (Agdia, USA) was negative. To determine the causal agent, we applied small RNA sequencing technology (1–3). Small RNA libraries were prepared as described previously (4) and sequenced using the Illumina HiSeq 2000. The sRNA sequences were assembled using the bioinformatics pipeline (3) without presubtraction of the host RNAs due to the unavailability of the zucchini squash genome sequence. Based on their strong sequence identities to the TSV okra Indian isolate (FJ561302, FJ561303, and FJ561301), the contigs were assembled into three genomic RNAs. Complete TSV genomic sequences were obtained after sequencing additional reverse transcription PCR (RT-PCR) products covering sequence gaps. The 5′ and 3′ terminal sequences in three TSV genomic RNAs were determined using rapid amplification of cDNA ends (RACE) (Ambion, USA). To facilitate 5′ adaptor ligation in RACE, the 5′ cap was removed by tobacco acid phosphatase (Epicentre, USA) to generate a 5′-monophosphate. The RT-PCR products were cloned into the TOPO-TA vector (Life Technologies, USA) and sequenced with Sanger sequencing. The complete TSV genome (isolate FL13-07) comprises three RNAs with 3,525, 2,898, and 2,211 nucleotides (nt) for RNA1, RNA2, and RNA3, respectively. RNA1 contains a single open reading frame (ORF) encoding a replicase (1,092 amino acids [aa]). RNA2 has two ORFs, with 2a encoding RNA-dependent RNA polymerase (790 aa) and 2b encoding a protein (199 aa) with an unknown function. RNA3 contains two ORFs encoding a movement protein (290 aa) and a coat protein (238 aa). BLASTn searches to the NCBI databases revealed that TSV_FL13-07 shares the highest sequence identities (99%) in RNA1 to several TSV isolates from India (pumpkin, GenBank accession no. FJ561299; okra, FJ561302; okra, KF264471; sunflower, KF264472; sunflower, KF264473; sunflower, KF264474; and soybean, KJ825823) but only 87 to 88% identities to several isolates in the United States (tobacco, JX073656; tobacco, U80934; and soybean, FJ403375). Similarly, RNA2 shares the highest sequence identity (98%) to two Indian isolates (okra, FJ561303; and pumpkin, FJ561300) but only 83 to 84% identity to several known U.S. isolates (soybean, FJ403376; tobacco, JX073657; and *Nicotiana*, U77738) and 80 to 88% to other isolates in Australia (*Verbesina encelioides*, JX463338; and *Helianthus annuus*, JX463335). For RNA3, a higher sequence identity (98% to 99%) was again shared with those isolates from India (squash, FJ655170; and *Vigna unguiculata*, FJ655171), with only 91% to an Australian isolate (*Verbesina encelioides*, JX463339) and 90% to two U.S. isolates (tobacco, JX073658; and soybean, FJ403377). In summary, sequence analyses indicated that TSV_FL13-07 represents an emerging genotype in the United States that shares only <90% sequence identities to other known U.S. isolates.

### Nucleotide sequence accession numbers.

The nucleotide sequences of TSV_FL13-07 (RNA1, RNA2, and RNA3) have been deposited in GenBank under the accession numbers KM504246, KM504247, and KM504248, respectively.
